# The Neuroprotective Effects of Arecae Pericarpium against Glutamate-Induced HT22 Cell Cytotoxicity

**DOI:** 10.3390/cimb44120402

**Published:** 2022-11-27

**Authors:** Yun Hee Jeong, You-Chang Oh, Tae In Kim, Jong-Sup Bae, Jin Yeul Ma

**Affiliations:** 1 Korean Medicine (KM)-Application Center, Korea Institute of Oriental Medicine, 70, Cheomdanro, Dong-gu, Daegu 41062, Republic of Korea; 2 College of Pharmacy, Research Institute of Pharmaceutical Sciences, Kyungpook National University, 80, Daehak-ro, Buk-gu, Daegu 41566, Republic of Korea

**Keywords:** Arecae Pericarpium, neuroprotection, antioxidant, phosphoinositide 3-kinase, protein kinase B, nuclear factor erythroid 2-related factor 2

## Abstract

Arecae Pericarpium has been found to exert anti-migraine, antidepressant, and antioxidative effects. However, the mechanisms involved are unclear. This study explored the possibility that Arecae Pericarpium ethanol extract (APE) exerts neuroprotective effects against oxidative stress-induced neuronal cell death. Since glutamate excitotoxicity has been implicated in the pathogenesis and development of several neurodegenerative disorders, we explored the mechanisms of action of APE on oxidative stress-induced by glutamate. Our results revealed that pretreatment with APE prevents glutamate-induced HT22 cell death. APE also reduced both the levels of intracellular reactive oxygen species and the apoptosis of cells, while maintaining glutamate-induced mitochondrial membrane potentials. Western blotting showed that pretreatment with APE facilitates the upregulation of phosphoinositide 3-kinase (PI3K)/protein kinase B (Akt) phosphorylation; the nuclear translocation of nuclear factor erythroid 2-related factor 2 (Nrf-2); and the production of antioxidant enzymes, including catalase, glutamate-cysteine ligase catalytic subunits, NAD(P)H quinone oxidoreductase 1, and heme oxygenase (HO)-1. The administration of LY294002, a PI3K/Akt inhibitor, attenuated the neuroprotective effects of APE on oxidative stress-induced neuronal cell damage. This allowed us to infer that the protective effects of APE on oxidative damage to cells can be attributed to the PI3K/Akt-mediated Nrf-2/HO-1 signaling pathway.

## 1. Introduction

Neuronal cell death plays an important role in the development of neuropathic diseases, and research has demonstrated that neurotransmitter imbalance is the primary cause of synaptic loss and degenerative cell death [1]. Within the central nervous system (CNS), glutamate is the principal endogenous neurotransmitter. However, excessive glutamate production or release can occur under certain pathological conditions, causing neuronal damage and death. This phenomenon has been found to contribute to many neurodegenerative diseases, including Alzheimer’s, Parkinson’s, and Huntington’s disease [2,3,4,5]. Glutamate is also known to trigger oxidative stress-mediated apoptosis in excitotoxic conditions [6]. High concentrations of glutamate increase the expression of both radical and non-radical reactive oxygen species (ROS). Glutamate-induced ROS production results in an extensive oxidative stress response, which, in turn, leads to mitochondrial dysfunction, cell damage, and death [7,8]. Therefore, the regulation of ROS production is a key target for the treatment or prevention of neurodegenerative diseases.

Nuclear factor erythroid 2-related factor 2 (Nrf-2) is a transcription factor that regulates the expression of many antioxidant enzymes, including catalase, glutamate-cysteine ligase catalytic (GCLC) subunits, NAD(P)H quinone oxidoreductase 1 (NQO1), and heme oxygenase (HO)-1. During oxidative stress stimulation, Nrf-2 translocates into the nucleus, binds with the antioxidant response element (ARE), and activates the production of antioxidant enzymes in neurons [9]. Several previous studies have shown that the nuclear translocation of Nrf-2 is closely related to phosphoinositide 3-kinase (PI3K)/protein kinase B (Akt) signaling, and activation of this signaling pathway can diminish oxidative stress and maintain mitochondrial integrity [9,10,11,12].

Arecae Pericarpium (AP) is derived from the seed husks of *Areca catechu* Linné (Palmae) and is a popular medicinal herb in Africa and the Asia-Pacific region. It is traditionally used to treat parasitic diseases, dyspepsia, and abdominal pain [13]. Previous studies have found *AP* to have wound healing, anti-migraine, antidepressant, anti-hypoglycemic, and antioxidant effects [14,15,16,17,18]. However, the physiological mechanisms of Arecae Pericarpium ethanol extract (APE) against glutamate-mediated neurotoxicity are yet to be clarified. In the present study, we investigated whether APE was able to protect hippocampal neurons against oxidative stress-induced apoptosis and cell death through the expression of Nrf-2/ARE-dependent antioxidant enzymes via activation of the PI3K/Akt pathway.

## 2. Materials and Methods

### 2.1. Chemicals and Reagents

Dulbecco’s Modified Eagle Medium (DMEM) was acquired from Welgene (Gyeongsan, Korea). Fetal bovine serum (FBS) and antibiotics were purchased from Hyclone (Logan, UT, USA). Bovine serum albumin (BSA) and dimethyl sulfoxide (DMSO) were purchased from Sigma–Aldrich (St. Louis, MO, USA). A cell-counting kit (CCK) was obtained from Dojindo Molecular Technologies, Inc. (Kumamoto, Japan). 2,7-dichlorodihydrofluorescein diacetate (H_2_DCFDA) was purchased from Invitrogen (Carlsbad, CA, USA) and an annexin V-FITC/propidium iodide (PI) apoptosis detection kit was obtained from BD Biosciences (Franklin Lakes, NJ, USA). The chloride salt, JC-1, was acquired from Biotium (Hayward, CA, USA). The primary antibodies and horseradish peroxidase (HRP)-conjugated secondary antibodies used in our Western blotting were purchased from Cell Signaling Technology, Inc. (Boston, MA, USA), Thermo Scientific (Rockford, IL, USA), and Santa Cruz Biotechnology (Santa Cruz, CA, USA).

### 2.2. Preparation of APE

AP was obtained from YeongcheonHyundai Herbal Market (Yeongcheon, Korea) and deposited in the herbal bank of the Korean Medicine Application Center, Korea Institute of Oriental Medicine (Daegu, Korea). The origins of the AP samples were assessed taxonomically by Professor Ki Hwan Bae of the College of Pharmacy, Chungnam National University (Daejeon, Korea). AP was extracted in 70% ethanol (50 g/ 390 mL) at 40 °C in a shaking incubator (100 rpm) for 24 h. The extract solution was filtered using 150 mm filter paper (Whatman, Piscataway, NJ, USA) and the filtrate was then concentrated in a vacuum evaporator (Buchi, Tokyo, Japan). Samples were freeze-dried and stored in a desiccator at −20 °C until use. For application to cells, APE lyophilized powder was dissolved using 50% DMSO (50% distilled water). The final DMSO concentration of each APE sample applied to cells is 0.005–0.1%.

### 2.3. Cell Culture and APE Treatment

The HT22 mouse hippocampal neuronal cell line was purchased from the American Type Culture Collection (Manassas, VA, USA) and grown in a DMEM medium that contains 10% of FBS and 1% of antibiotics at 37 °C in a 95% air/5% CO_2_ incubator. The cells were treated with 5 mM glutamate in the presence or absence of APE (10, 50, 100, or 200 μg/mL).

### 2.4. Cell Viability Test

HT22 cells were seeded into 96-well culture plates at a density of 5 × 10^3^ cells/ well for 24 h, with 100 μL of complete DMEM. The cells were treated with APE at concentrations of 10, 50, 100, or 200 μg/mL for 2 h before exposure to 5 mM glutamate. After 24 h, 10 μL of CCK solution was added to each well, and they were incubated for 1 h. The absorbance was measured at 450 nm using a microplate reader (SpectraMax i3, Molecular Devices, San Jose, CA, USA).

### 2.5. Lactate Dehydrogenase (LDH) Release Assay

The release of LDH in the culture medium was determined using an LDH assay kit in accordance with the supplier’s instructions. The HT22 cells were treated with APE at concentrations of 10, 50, 100, or 200 μg/mL for 2 h before exposure to 5 mM glutamate for 24 h. After stimulation with glutamate, the culture medium was transferred to a new 96-well plate. The reaction solution was added to each well, and LDH activity was examined using a microplate reader at 450 nm. LDH release was expressed as a percentage of that observed in the control cells.

### 2.6. Measurement of Intracellular ROS Levels

Intracellular ROS levels were measured using H_2_DCFDA. The HT22 cells were treated with APE at concentrations of 10, 50, 100, or 200 μg/mL for 2 h before exposure to 5 mM glutamate for 6 h. After stimulation with glutamate, the cells were stained and incubated in darkness with 10 μM H_2_DCFDA for 30 min. Fluorescence (an excitation wavelength of 485 nm and an emission wavelength of 525 nm) was estimated using a microplate reader. Representative fluorescence images were obtained using a fluorescence microscope (Eclipse Ti, Nikon, Tokyo, Japan).

### 2.7. Analysis of Mitochondrial Membrane Potential (MMP)

The fluorescent probe, JC-1, was used to detect MMP, according to the manufacturer’s instructions. The HT22 cells were treated with APE at concentrations of 10, 50, 100, or 200 μg/mL for 2 h before exposure to 5 mM glutamate for 24 h. Then, 5 μg/mL JC-1 solution was prepared prior to use, and cells were stained at 37 °C in darkness for 15 min. They were then rinsed twice with PBS, and fluorescence signals were measured by spectrofluorometry (SpectraMax i3) at 490 nm for excitation, 530 nm for green (monomer form) fluorescence, 520 nm for excitation, and 590 nm for red (aggregate form) fluorescence. Representative fluorescence images were obtained using a fluorescence microscope.

### 2.8. Immunoblot Analysis

The HT22 cells were treated with APE or LY294002 for 2 h before exposure to 5 mM glutamate. After stimulation with glutamate, the cells were lysed using a radioimmunoprecipitation assay lysis buffer (Millipore, Bedford, MA, USA) by adding a protease and phosphatase inhibitor cocktail (Roche, Basel, Switzerland). The total proteins (20 μg) from the supernatant were resolved by sodium dodecyl sulfate-polyacrylamide gel electrophoresis and transferred onto a polyvinylidene difluoride membrane. The membrane was blocked with 3% BSA at room temperature (RT) for 1 h and then incubated overnight with primary antibodies against the target protein. The membranes were then washed and incubated with HRP-conjugated secondary antibodies for 1 h at RT. The relative intensity of the protein expression was quantitated using the ChemiDoc™ Touch Imaging System (Bio-Rad, Hercules, CA, USA). The relative protein expression was determined using Image J software (version 1.53k), with normalization to the control value. Table 1 details the primary and secondary antibodies used.

### 2.9. Nuclear and Cytosolic Protein Extraction

Cytosolic and nuclear proteins were fractionated using NE-PER Nuclear and Cytoplasmic Extraction Reagents (Thermo Scientific), according to the manufacturer’s instructions.

### 2.10. Flow Cytometry Assessment of Cell Death

Glutamate-induced apoptotic cell death, and its inhibition by APE, were determined using the annexin V-FITC/PI Apoptosis Detection Kit. The HT22 cells were treated with APE at concentrations of 50, 100, or 200 μg/mL for 2 h before exposure to 5 mM glutamate for 24 h. The cells were harvested and stained with 5 μL of annexin V-FITC/PI at 37 °C in darkness for 15 min. They were then analyzed using flow cytometry (FACS Calibur, BD Biosciences, Franklin Lakes, NJ, USA).

### 2.11. Statistical Analysis

All of the experiments were performed three times and the data of all repetitions of each experiment were collated and expressed as means ± standard error of the mean. Statistical tests were conducted using GraphPad Prism version 5.02 (GraphPad Software, Inc., San Diego, CA, USA). Data were analyzed by one-way analysis of variance, followed by Dunnett’s test, after comparing each sample. Statistical significance was set at *p* <0.05.

## 3. Results

### 3.1. Neuroprotective Effects of APE on Glutamate-Induced HT22 Cell Toxicity

**Figure 1:**
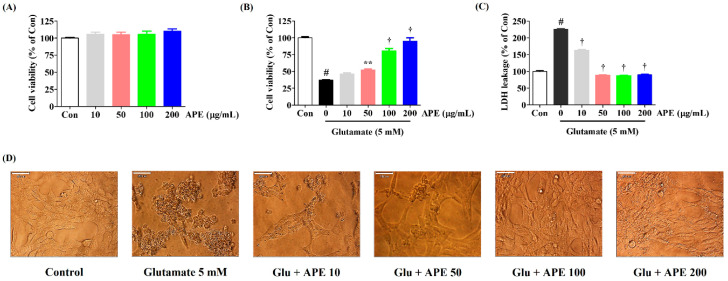
Effects of Arecae Pericarpium ethanol extract on glutamate-induced cytotoxicity in HT22 cells. (**A**) HT22 cells were incubated with APE at concentrations of 10, 50, 100, or 200 μg/mL. (**B**–**D**) After APE pretreatment, the HT22 cells were stimulated with glutamate (5 mM). (**D**) The images represent the three independent experiments, shown at 1000× magnification. Scale bar = 20 μm. Control was non-treated cells. Data were presented as mean ± standard error of the mean. Statistical significance was set at # *p* < 0.05 (vs. control), ** *p* < 0.01, and † *p* < 0.001 (vs. glutamate). APE, Arecae Pericarpium ethanol extract; Con, control; Glu, glutamate; LDH, lactate dehydrogenase.

We measured the effects of APE on HT22 cell viability using a CCK assay. As shown in Figure 1A, treatment with APE alone produced no cytotoxicity at any dosage up to 200 μg/mL. Thus, concentrations of 200 μg/mL and below were used in the rest of the experiments as these dosages were safe and did not affect the survival of HT22 cells.

To identify possible protective effects of APE against glutamate-induced neurotoxicity in HT22 cells, CCK and LDH assays were performed. Our results showed a decrease in cell viability of approximately 63% in cells treated with glutamate compared to the control cells (Figure 1B). Pretreatment with APE markedly increased the cell viability of glutamate-treated HT22 cells in a concentration-dependent manner (Figure 1B). At 200 μg/mL of APE, cell viability was similar to that of the control cells. An LDH cell cytotoxicity assay was performed to support the cell viability results. As shown in Figure 1C, LDH release in the cells treated with glutamate alone increased to 225% compared with control cells. However, pretreatment with different concentrations of APE (50, 100, or 200 μg/mL) reduced LDH release to 88%, 87%, and 90%, respectively of that seen in the control cells (Figure 1C). Additionally, changes in cell morphology were observed by microscopy. Compared with the control, glutamate-treated cells were extensively damaged and lacked axons and dendrites (Figure 1D). However, when the cells were treated with 200 μg/mL APE, they recovered to a state comparable to that of the control cells (Figure 1D).

### 3.2. Effects of APE on Glutamate-Induced ROS Generation

**Figure 2:**
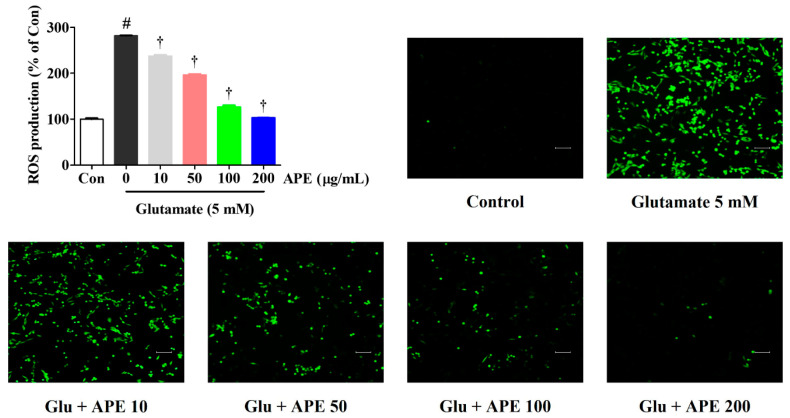
Effects of Arecae Pericarpium ethanol extract on glutamate-induced intracellular ROS production in HT22 cells. Cells were pretreated with APE at concentrations of 10, 50, 100, or 200 μg/mL and then with 5 mM glutamate. H_2_DCFDA (20 μM), an oxidation-sensitive fluorescent dye, was used to assess ROS levels. The expression of ROS was determined using a fluorescence microscope and fluorescence microplate reader. Scale bar = 100 μm. Control was non-treated cells. All experiments were repeated at least three times, and similar results were obtained. Data were presented as mean ± standard error of the mean. Statistical significance was set at # *p* < 0.05 (vs. control) and † *p* < 0.001 (vs. glutamate). APE, Arecae Pericarpium ethanol extract; Con, control; Glu, glutamate; ROS, reactive oxygen species.

Using an H_2_DCFDA fluorescence assay, we explored the ability of APE to counteract intracellular ROS production, which is associated with neuronal dysfunction and cell death. Our results show a significant increase in ROS production after treatment with 5 mM glutamate compared with non-treated control cells. However, pretreatment with APE significantly reduced ROS generation in a concentration-dependent manner (Figure 2).

### 3.3. Effects of APE on MMP in Glutamate-Treated HT22 Cells

**Figure 3:**
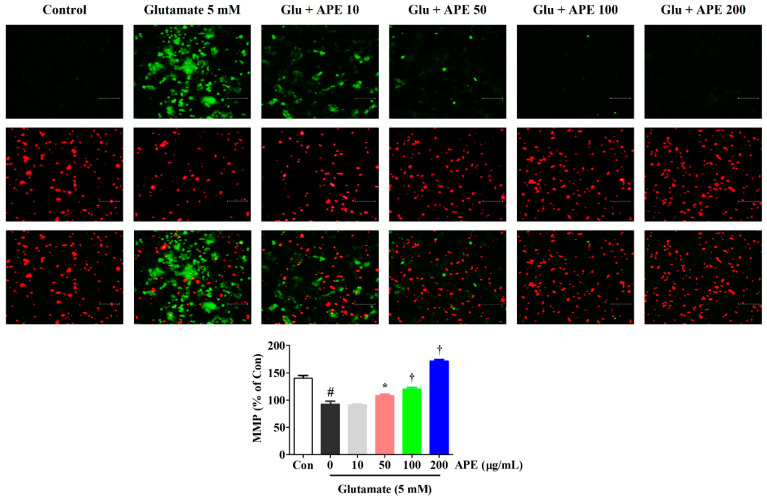
Effects of Arecae Pericarpium ethanol extract on glutamate-induced mitochondrial dysfunction in HT22 cells. MMP was assessed via microscopy using JC-1 staining. The images represent the three independent experiments, shown at 200× magnification. Scale bar = 100 μm. Red fluorescence indicated normal MMP and green fluorescence, damaged mitochondria with MMP loss. The histogram shows the red/green fluorescence intensity ratio. Control was non-treated cells. Data were presented as mean ± standard error of the mean. Statistical significance was set at # *p* < 0.05 (vs. control), * *p* < 0.05, and † *p* < 0.001 (vs. glutamate). APE, Arecae Pericarpium ethanol extract; Con, control; Glu, glutamate; MMP, mitochondrial membrane potential.

To determine whether the neuroprotective effects of APE are due to the preservation of mitochondrial function in glutamate-treated cells, we examined MMP using a JC-1 fluorescence staining assay. JC-1 aggregates in normal mitochondria and exhibits red fluorescence, which is indicative of normal MMP. Green fluorescence is exhibited when mitochondria have depolarized during apoptotic cell death. As shown in Figure 3, the control cells predominantly exhibited red fluorescence, with little green fluorescence. However, cells treated with glutamate for 24 h showed an increase in green fluorescence and concomitant disappearance of red fluorescence. APE treatment (50, 100, or 200 μg/mL) of the glutamate-damaged cells produced a decrease in green fluorescence and an increase in red fluorescence intensity. In particular, at a concentration of 200 μg/mL, APE caused a JC-1 distribution similar to that in control cells (Figure 3). To quantify these MMP changes, the red/green fluorescence intensities were measured using a fluorescence plate reader. Compared to the control cells, a significant reduction of red/green fluorescence was observed in glutamate alone-treated cells. However, pretreatment with APE dose-dependently elevated the red/green fluorescence intensity.

### 3.4. Flow Cytometry Assessment of Apoptosis Prevention by APE in HT22 Cells

**Figure 4:**
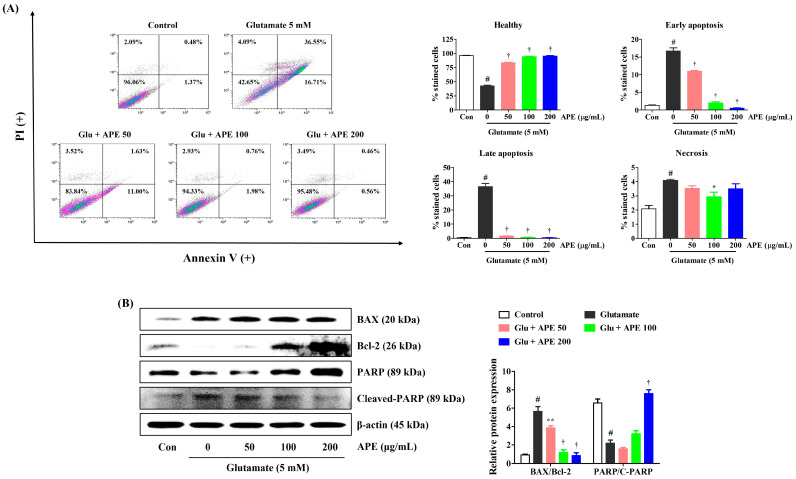
Effects of Arecae Pericarpium ethanol extract on glutamate-induced apoptosis in HT22 cells. Cells were pretreated with APE at concentrations of 50, 100, or 200 μg/mL and then treated with glutamate (5 mM). (**A**) Apoptosis of HT22 cells was evaluated using flow cytometry. The image on the top right shows the percentage of healthy, early apoptotic, late apoptotic, and necrotic cells for each treatment group. (**B**) The expression levels of BAX, Bcl-2, PARP, and cleaved-PARP were determined by Western blot analysis. Control was non-treated cells. Blot images represent the three independent experiments. Data were presented as mean ± standard error of the mean. Statistical significance was set at # *p* < 0.05 (vs. control), * *p* < 0.05, ** *p* < 0.01, and † *p* < 0.001 (vs. glutamate). APE, Arecae Pericarpium ethanol extract; BAX, Bcl-2-associated X; Bcl-2, B-cell lymphoma 2; Con, control; Glu, glutamate; PI, propidium iodide; PARP, Poly(ADP-ribose) polymerase.

To examine whether APE protects against glutamate-induced apoptosis, HT22 cells were incubated with APE for 2 h and treated with 5 mM glutamate for 24 h. After exposure to glutamate, the percentage of apoptotic cells increased to about 57% (Figure 4A). In contrast, pretreatment with APE resulted in a marked reduction in the apoptotic population (Figure 4A). We further assessed the effects of APE on the expression of apoptosis-related proteins in glutamate-exposed HT22 cells. As shown in Figure 4B, treatment with glutamate increased the expression of apoptotic markers in HT22 cells. APE pretreatment significantly reduced the expression of cleaved poly (ADP-ribose) polymerase (PARP), a caspase-independent pro-apoptotic factor. Furthermore, it enhanced the levels of anti-apoptotic factors, such as B-cell lymphoma 2 (Bcl-2) and PARP, compared with that in cells treated with glutamate alone (Figure 4B). These results indicate that the neuroprotective effects of APE pretreatment are partially achieved by the reduction of glutamate-induced apoptotic cell death.

### 3.5. APE Activation of Antioxidant Enzyme Protein Expression

**Figure 5:**
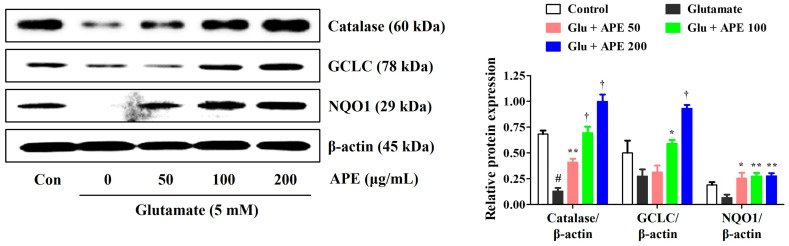
Effects of Arecae Pericarpium ethanol extract on the activation of antioxidant enzymes in glutamate-exposed HT22 cells. The cells were incubated with 5 mM glutamate with or without *APE*. The expression levels of catalase, GCLC subunits, and NQO1 were determined via Western blot analysis. Control was non-treated cells. Blot images represent the three independent experiments. Data were presented as mean ± standard error of the mean. Statistical significance was set at # *p* < 0.05 (vs. control), * *p* < 0.05, ** *p* < 0.01, and † *p* < 0.001 (vs. glutamate). APE, Arecae Pericarpium ethanol extract; Con, control; GCLC, glutamate-cysteine ligase catalytic; Glu, glutamate; NQO1, NAD(P)H quinone oxidoreductase 1.

To elucidate the antioxidative mechanisms of APE against glutamate-induced cell injury, we measured the production of the antioxidant enzymes, catalase, GCLC, and NQO1, by Western blot. Our results showed that glutamate significantly reduces the expression of catalase, GCLC, and NQO1. In contrast, pretreatment with APE notably enhanced the levels of these enzymes (Figure 5). These data suggest that the expression of catalase, GCLC, and NQO1 is implicated in the preventive action of APE against glutamate-induced oxidative stress.

### 3.6. APE Enhancement of the Expression of PI3K/Akt and Nrf-2/HO-1 Signaling Pathways

**Figure 6:**
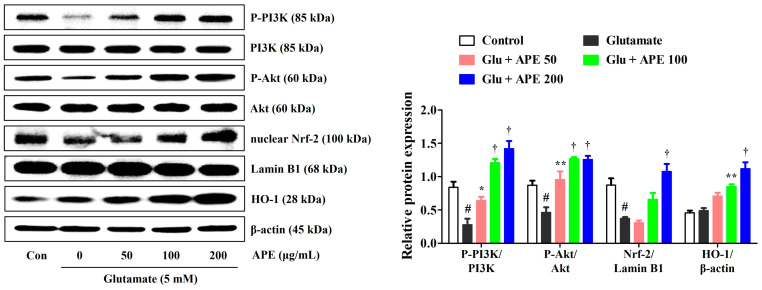
Effects of Arecae Pericarpium ethanol extract on the phosphorylation of PI3K and protein kinase B, the nuclear translocation of Nrf-2, and the expression of HO1 in glutamate-exposed HT22 cells. Cells were pretreated with APE at concentrations of 50, 100, or 200 μg/mL and then treated with glutamate (5 mM). Control was non-treated cells. Blot images represent the three independent experiments. Data were presented as mean ± standard error of the mean. Statistical significance was set at # *p* < 0.05 (vs. control), * *p* < 0.05, ** *p* < 0.01, and † *p* < 0.001 (vs. glutamate). Akt, protein kinase B; APE, Arecae Pericarpium ethanol extract; Con, control; Glu, glutamate; HO1, heme oxygenase-1; Nrf-2, nuclear factor erythroid 2-related factor 2; PI3K, phosphoinositide 3-kinase.

To investigate the mechanisms underlying the prevention of glutamate-induced apoptosis, we analyzed the effects of APE on the expression of PI3K/Akt and Nrf-2/HO-1 in HT22 cells. A previous study has found the PI3K/Akt signaling pathway to be important for the activation and nuclear translocation of Nrf-2 [19]. Activation of the PI3K/Akt/Nrf-2 signaling pathways has also been shown to be closely related to neuroprotective activity [9]. Therefore, the effects of APE on the expression of PI3K/Akt, Nrf-2, and antioxidant enzymes were determined by Western blot. As shown in Figure 6, pretreatment with APE dramatically increased the phosphorylation of PI3K/Akt compared with that of cells treated with glutamate alone. Additionally, APE treatment increased nuclear translocation of Nrf-2 and its downstream HO-1 expression in a concentration-dependent manner (Figure 6).

### 3.7. LY294002 Inhibition of the Neuroprotective Effects of APE

**Figure 7:**
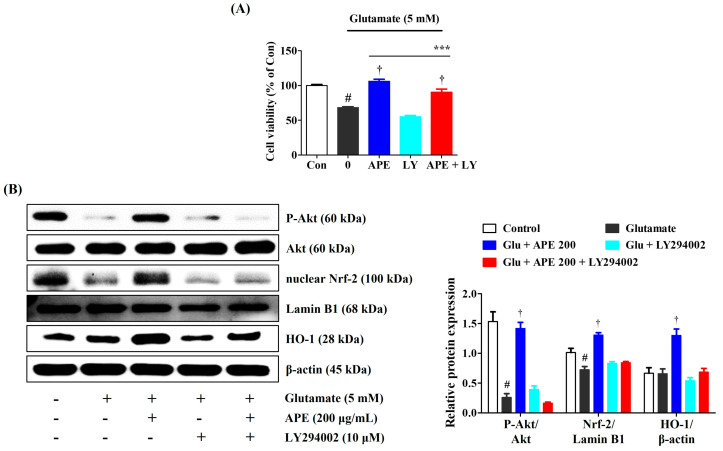
The suppressive effects of LY294002 on the neuroprotective action of Arecae Pericarpium ethanol extract. HT22 cells were incubated with or without APE combined with LY294002 before glutamate treatment. (**A**) Cell viability and, (**B**) Activation of protein kinase B, Nrf-2, and HO1 were assessed using a cell-counting kit assay and Western blot analysis, respectively. Control was non-treated cells. Blot images represent the three independent experiments. Data were presented as mean ± standard error of the mean. Statistical significance was set at # *p* < 0.05 (vs. control), † *p* < 0.001 (vs. glutamate), and *** *p* < 0.001 (vs. APE). Akt, protein kinase B; APE, Arecae Pericarpium ethanol extract; Con, control; Glu, glutamate; HO, heme oxygenase; LY, LY294002; Nrf-2, nuclear factor erythroid 2-related factor 2.

Nrf-2/HO-1 signaling might be activated by the PI3K/Akt pathway. The PI3K inhibitor, LY294002, was used to identify whether activation of the PI3K/Akt pathway mediates the protective effects of APE in glutamate-treated HT22 cells. As shown in Figure 7, combining APE with LY294002 markedly decreased the neuroprotective effects of APE on glutamate-induced cell death. Furthermore, LY294002 significantly exacerbated APE-mediated phosphorylation of PI3K/Akt and reduced the activation of Nrf-2/HO-1 signaling (Figure 7).

## 4. Discussion

Aberrant glutamatergic neurotransmission has been reported to be associated with the pathological processes of various neurodegenerative diseases [20,21,22]. Glutamate is the principal endogenous neurotransmitter in the CNS. However, excessive glutamate causes neuronal damage and death [2,3,4,5]. The production of ROS is a major marker of glutamate-induced neurotoxicity [23]. Thus, ROS-induced oxidative stress plays an important role in the neuronal cell damage and death associated with the pathophysiology of neurodegenerative diseases [7,8]. Therefore, the modulation of glutamate for the prevention of ROS-induced oxidative stress is a promising strategy for combating excitotoxicity.

There is evidence that extracts from the medicinal plant, Arecae Pericarpium has various therapeutic effects, including wound healing, anti-migraine, anti-depressant, antioxidative, and hypoglycemic [14,15,16,17,18]. Nevertheless, there has been no previous research on the neuroprotective effects of APE against glutamate-mediated neurotoxicity and the mechanisms involved. In the present study, we examined the therapeutic potential and underlying mechanisms of APE against neuroexcitotoxicity induced by glutamate imbalance.

We found that pretreatment with APE protected HT22 cells from glutamate-induced cytotoxicity. This was evidenced by high cell viability and low LDH release (Figure 1). APE pretreatment also minimized glutamate-induced cell damage through the reduction of intracellular ROS levels (Figure 2). The accumulation of ROS following glutamate stimulation is closely related to changes in mitochondrial permeability and diffusion of MMP and has been linked with many apoptotic phenomena [24,25]. We found that APE treatment improves glutamate-induced loss of MMP in a concentration-dependent manner (Figure 3).

We used flow cytometry to investigate cell death resulting from glutamate-induced oxidative stress. We found that pretreatment with APE notably reduced apoptosis in glutamate-exposed HT22 cells (Figure 4). Consistent with these findings, APE resulted in significant inhibition of mitochondrial apoptotic factors, including Bcl-2-associated X (BAX), cleaved-PARP, and cleaved-caspase-3; as well as significant elevation of anti-apoptotic factors, including Bcl-2 and PARP (Figure 4). These results suggest that APE protects neurons from glutamate-mediated apoptosis by regulating MMP and the generation of ROS.

To further examine the mechanisms underlying the neuroprotective effect of APE, Nrf-2 activation and the production of downstream antioxidant enzymes were investigated. Nrf-2 is an important transcription factor responsible for activating the transcription of several antioxidant enzymes, including catalase, GCLC, NQO1, and HO-1. This produces a notable counteractive antioxidative response to the escalation of ROS-induced oxidative stress. In its unstimulated state, Nrf-2 is present in the cytoplasm bound to Kelch-like ECH-associated protein 1 (KEAP1). After activation by oxidative stress stimulants, Nrf-2 dissociates from KEAP1, translocates to the nucleus, binds to ARE, and triggers the production of antioxidant enzymes in neuronal cells. Previous studies have suggested that Nrf-2-mediated pathways exert neuroprotective effects by inhibiting oxidative stress and maintaining the integrity of the mitochondria [11,12]. Since Nrf-2/ARE signaling primarily regulates Nrf-2 nuclear translocation through the activation of upstream signaling in the PI3K/Akt pathway [9,10,11,12], we also investigated whether APE exerts its neuroprotective effects through activation of the PI3K/Akt-mediated Nrf-2 signaling pathway. Our results indicated that pretreatment with APE strongly promotes nuclear translocation of Nrf-2 and the expression of antioxidant enzymes, including catalase, GCLC, NQO1, and HO-1, in glutamate-treated HT22 cells (Figure 5 and Figure 6). APE also strongly upregulates the expression of PI3K and P-Akt (Figure 6). Furthermore, the addition of LY294002, a PI3K pathway inhibitor, nullified the inhibitory effects of APE on neurocytotoxicity, significantly blocked the phosphorylation of PI3K/Akt, and suppressed downstream Nrf-2/HO-1 activation in glutamate-treated HT22 cells (Figure 7). Therefore, it appears that APE exerts its neuroprotective effects through the activation of the PI3K/Akt-dependent Nrf-2/HO-1 signaling pathway.

## 5. Conclusions

In summary, the current study demonstrates that pretreatment with APE can protect neuronal cells from glutamate-induced oxidative stress. We have demonstrated remarkable attenuation by APE of the neuronal damage caused by intracellular oxidation/antioxidant imbalance and apoptosis. We also found that APE exerts these neuroprotective effects through the activation of the PI3K/Akt-dependent Nrf-2/ARE signaling pathway. Our findings suggest that APE has the potential for use in the prevention and treatment of neurodegenerative diseases.

## Figures and Tables

**Table 1:** Primary and secondary antibodies use for Western blot analysis.

Antibody	Corporation	Product No.	RRID	Dilution Rate
BAX	Cell Signaling	#2772	AB_10695870	1:1000
Bcl-2	Cell Signaling	#3498	AB_1903907	1:1000
PARP	Cell Signaling	#9532	AB_659884	1:1000
Cleaved-PARP	Cell Signaling	#9548	AB_2160592	1:1000
β-actin	Cell Signaling	#4970	AB_2223172	1:1000
Catalase	Cell Signaling	#14097	AB_2798391	1:1000
GCLC	Thermo Fisher	#PA5-87854	AB_2804457	1:1000
NQO1	Santa Cruz	#sc-32793	AB_628036	1:1000
P-PI3K	Cell Signaling	#17366	AB_2895293	1:1000
PI3K	Cell Signaling	#4257	AB_659889	1:1000
P-Akt	Cell Signaling	#4060	AB_2315049	1:1000
Akt	Cell Signaling	#4691	AB_915783	1:1000
Nrf-2	Cell Signaling	#12721	AB_2715528	1:1000
Lamin B1	Cell Signaling	#13435	AB_2737428	1:1000
HO-1	Cell Signaling	#82206	AB_2799989	1:1000
2nd anti-mouse	Cell Signaling	#7076	AB_330924	1:5000
2nd anti-rabbit	Cell Signaling	#7074	AB_2099233	1:5000

## Data Availability

The data are contained within the article.
